# Supplementary material for: The Diagnostic Performance of Tumor Stage on MRI for Predicting Prostate Cancer-Positive Surgical Margins: A Systematic Review and Meta-Analysis
Source: Diagnostics (Basel). 2023 Jul 27;13(15):2497. doi: 10.3390/diagnostics13152497 (PMC10417235; doi:10.3390/diagnostics13152497)
Supplement: Supplementary file 1 [file diagnostics-13-02497-s001.zip › diagnostics-2474808-supplementary.pdf]

PubMed search strategy
#1 Prostatic Neoplasms [MeSH Major Topic]
#2 Prostate Cancer [Title/Abstract]
#3 Prostatic carcinoma [Title/Abstract]
#4 Prostatic tumor [Title/Abstract]
#5 #1 OR #2 OR #3 OR #4
#6 Magnetic Resonance Imaging [MeSH Major Topic]
#7 MRI [Title/Abstract]
#8 #6 OR #7
#9 Margins of Excision [MeSH Major Topic]
#10 Surgical Margins [Title/Abstract]
#11 Resection Margins [Title/Abstract]
#12#9 OR #10 OR #11
#13 #5 AND #8 AND #12
#14 #13 AND ("2000/01/31"[Date - Publication] : "2022/12/31"[Date - Publication])

EMBASE search strategy
#1 'prostate cancer'/exp
#2 'prostate tumor'/exp
#3 prostate carcinoma:ti,ab,kw
#4 #1 OR #2 OR #3
#5 MRI:ti,ab,kw
#6 'magnetic resonance imaging':ti,ab,kw
#7 'nuclear magnetic resonance imaging'/exp
#8 #5 OR #6 OR #7
#9 'surgical margin'/exp
#10 'resection margins':ti,ab,kw
#11 'margins of excision':ti,ab,kw
#12 #9 OR #10 OR #11
#13 #4 AND #8 AND #12
#14 #4 AND #8 AND #12 AND [01-01-2000]/sd NOT [12-31-2022]

Cochrane search strategy
#1 MeSH descriptor: [Prostatic Neoplasms] explode all trees
#2 Prostate Prostate Cancer or Prostatic carcinoma or Prostatic tumorCancer
#3 #1 or #2
#4 MeSH descriptor: [Magnetic Resonance Imaging] explode all trees
#5 MRI
#6 #4 or #5
#7 MeSH descriptor: [Margins of Excision] explode all trees
#8 Surgical Margins or Resection Margins
#9 #7 or #8
#10 #3 and #6 and #9 with Cochrane Library publication date Between Jan 2000 and Dec 2022
